# Pneumococcal colonization and invasive disease studied in a porcine model

**DOI:** 10.1186/s12866-016-0718-3

**Published:** 2016-06-08

**Authors:** Astrid de Greeff, Saskia van Selm, Herma Buys, José F. Harders-Westerveen, Rahajeng N. Tunjungputri, Quirijn de Mast, Andre J. van der Ven, Norbert Stockhofe-Zurwieden, Marien I. de Jonge, Hilde E. Smith

**Affiliations:** Central Veterinary Institute, part of Wageningen UR, Lelystad, The Netherlands; Laboratory of Paediatric Infectious Diseases, Department of Paediatrics, Radboud University Medical Center, Nijmegen, The Netherlands; Raboud Institute for Molecular Life Sciences, Nijmegen, The Netherlands; Department of Internal Medicine, Radboud University Medical Center, Nijmegen, The Netherlands

**Keywords:** Streptococcus pneumoniae, Animal model, Pigs, Colonization

## Abstract

**Background:**

*Streptococcus pneumoniae*, a Gram-positive bacterium carried in the human nasopharynx, is an important human pathogen causing mild diseases such as otitis media and sinusitis as well as severe diseases including pneumonia, meningitis and sepsis. There is a strong resemblance between the anatomy, immunology and physiology of the pig and human species. Furthermore, there are striking similarities between *S. suis* pathogenesis in piglets and *S. pneumoniae* pathogenesis in humans. Therefore, we investigated the use of piglets as a model for pneumococcal colonization and invasive disease.

**Results:**

Intravenous inoculation of piglets with an invasive pneumococcal isolate led to bacteraemia during 5 days, showing clear bacterial replication in the first two days. Bacteraemia was frequently associated with fever and septic arthritis. Moreover, intranasal inoculation of piglets with a nasopharyngeal isolate led to colonization for at least six consecutive days.

**Conclusions:**

This demonstrates that central aspects of human pneumococcal infections can be modelled in piglets enabling the use of this model for studies on colonization and transmission but also on development of vaccines and host-directed therapies. Moreover this is the first example of an animal model inducing high levels of pneumococcal septic arthritis.

## Background

*Streptococcus pneumoniae* is a Gram positive bacterium carried in the nasopharynx and spread by human-to-human transmission causing different diseases ranging from mild, such as otitis media and sinusitis, to severe diseases, including pneumonia, meningitis and sepsis [1, 2]. Furthermore, *S. pneumoniae* is an important cause of septic arthritis [3], and endocarditis [4]. Asymptomatic colonization of the nasopharynx is an essential prerequisite for pneumococcal disease. Worldwide about 40-90 % of children and 10 % of adults are colonized with *S. pneumoniae* [5]. Risk groups for pneumococcal disease are children under the age of 2 years, elderly people and immunocompromised patients [1]. Morbidity and mortality caused by *S. pneumoniae* infections are high, both in the developing and the developed world: annually 1.6 million people die worldwide of pneumococcal infections [6].

Since pigs are very similar to humans in terms of anatomy, immunology and physiology [7] they might be a good model to mimic human disease. More importantly, the porcine immune system closely resembles that of the human immune system for over 80 % of the analyzed parameters, whereas for mice this is less than 10 % [8]. For example the porcine complement activation is similar to that of humans in all pathways [9] and pigs have Th17 cells with functions similar to their human counterparts [10]. Moreover, the cardiovascular physiology of pigs and men are very similar, indicating that pig models could be used to study the effects of *S. pneumoniae* on the cardiovascular system. Pigs have been described as suitable models for human infectious diseases of different nature, like Gram-negative bacteria induced meningitis [11] and pneumonia [12], Gram-positive bacteria induced endocarditis [13], scabies infections [14] and fungal infections [15]. Moreover, the availability of a large animal model allows to do repeated measurements, for example colonization can be monitored in time without killing the animal.

Piglets are the natural host for *Streptococcus suis* infections. There are striking similarities between *S. suis* pathogenesis in piglets and *S. pneumoniae* pathogenesis in humans. Piglets develop severe disease like meningitis, sepsis, arthritis, endocarditis or pneumonia upon infection with *S. suis* [16]. *S. suis* is carried in the oropharynx, the bacterium colonizes the tonsil of piglets, similar to *S. pneumoniae* in children [17]. Furthermore, genetically *S. suis* and *S. pneumoniae* are closely related [18]. In this study, we investigate the use of piglets as a model for human colonization and invasive disease caused by *S. pneumoniae*. We demonstrate that the pig is a suitable model to study pneumococcal colonization and invasive disease that could potentially be used for the development of new vaccines and therapies.

## Results

### Bacteraemia

**Fig. 1:**
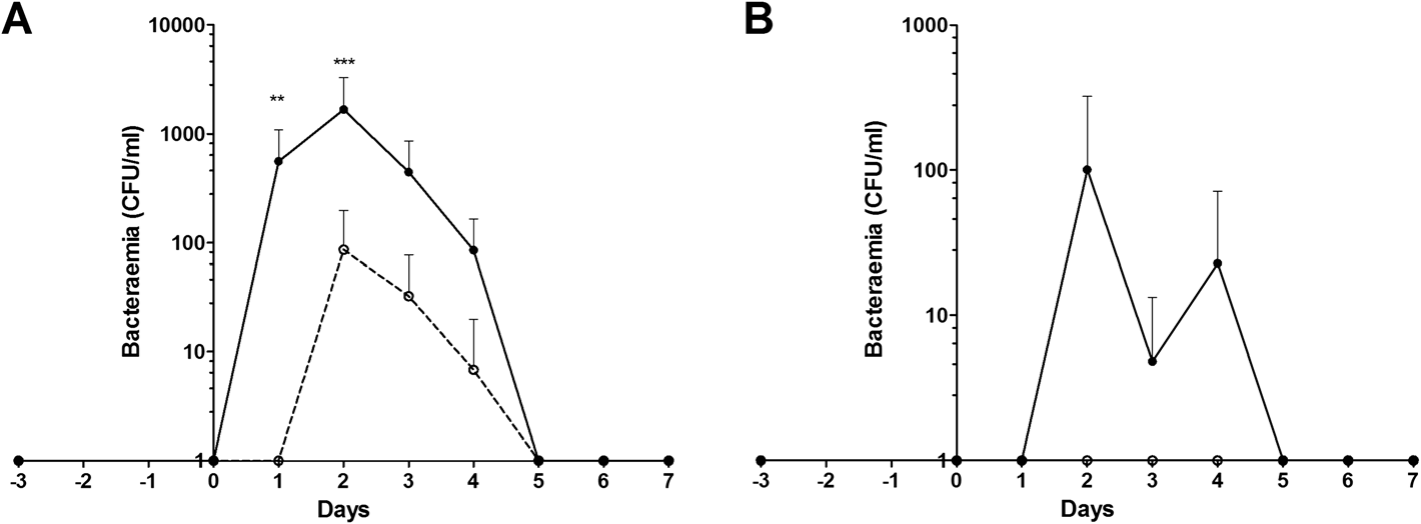
Bacteraemia in piglets due inoculation with *S. pneumoniae*. Bacteraemia was monitored on a daily base in piglets infected intravenously (Panel **a**) or intranasally (Panel **b**) with *S. pneumoniae* by plating EDTA blood on selective agar plates. Mean bacterial counts are depicted, error bars represent standard error of the mean. Open symbols represent piglets infected with a low dose, closed symbols piglets infected with a high dose. Each symbol represents 3 − 5 piglets, error bars show standard deviation. ** *p* < 0.01; *** *p* < 0.001 (ANOVA)

PBCN0214 is a serotype 8 strain, which was isolated from a patient with pneumonia, meningitis and sepsis without underlying co-morbidities. The piglets were inoculated intravenously, in the vena jugularis, with a high (*n* = 5) or a low dose (*n* = 5). All piglets, with the high dose as well as with the low dose, developed bacteraemia (Fig. 1a). Bacterial numbers increased during the first two days, indicating that pneumococci replicated in the blood. However, piglets inoculated with the low dose cleared the bacteria faster and reached a significantly lower number of colony forming units (CFU) in blood than piglets inoculated with the high dose (p < 0.01) (Fig. 1a). Interestingly, one of the intranasally inoculated piglets developed pneumococcal bacteraemia as well, clearly demonstrating that *S. pneumoniae* is able to breach host defence barriers in pigs after intranasal inoculation (Fig. 1b). Bacteria isolated from blood were confirmed to be *S. pneumoniae* using Matrix-Assisted Laser Desorption/Ionization–Time of Flight (MALDI-TOF).

### Oropharyngeal colonization

**Fig. 2:**
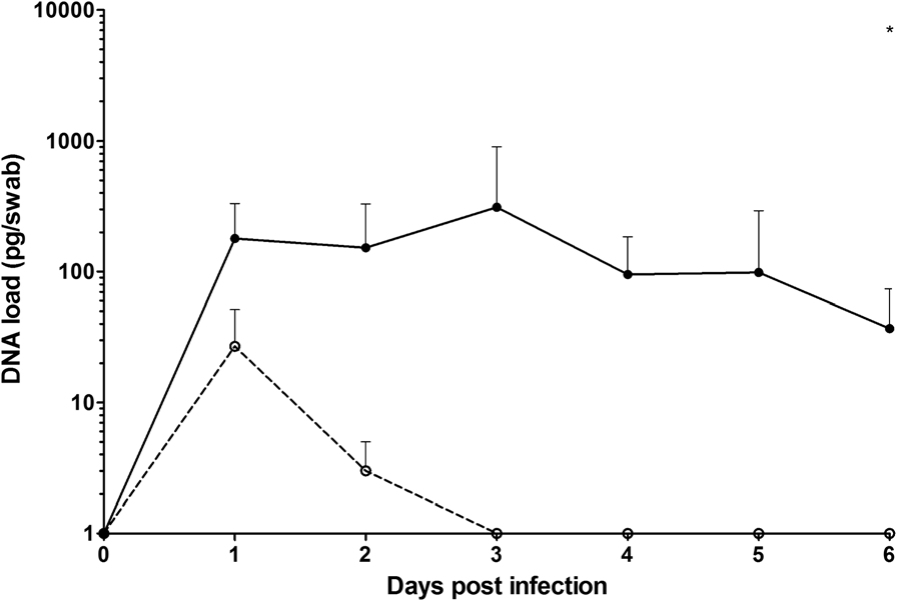
Colonization of *S. pneumoniae* in the oropharynx of intranasally inoculated piglets. Colonization with *S. pneumoniae* was detected using a pneumococcal specific qPCR. At day 0 prior to infection, no *S. pneumoniae* was detected. Open symbols represent piglets infected with a low dose, closed symbols piglets infected with a high dose. Each symbol represents 3 − 5 piglets, error bars show standard deviation. * *p* < 0.05 (ANOVA). 100 pg of DNA is equivalent to approximately 4 × 10^5^ CFUs

BHN418, a serotype 6B strain, has been frequently described in an experimental human carriage model [19, 20]. This strain was originally isolated from a colonized individual [21]. The piglets were inoculated intranasally with a high (*n* = 5) or a low dose (*n* = 5). Because selective culturing of *S. pneumoniae* using gentamycin was not possible in pigs due to overgrowth of many different organisms, the pneumococcal colonization of the oropharyngeal cavity was measured by polymerase chain reaction (PCR) on tonsil swab samples. The data (Fig. 2) show that the piglets inoculated intranasally with the highest dose, were colonized with *S. pneumoniae* throughout the experiment. Two piglets from this group were not (detectably) colonized at the end of the experiment. The oropharynx of all five piglets in this group was colonized for at least 5 days. Piglets intranasally inoculated with the low dose showed limited colonization. These data clearly showed that *S. pneumoniae* can colonize the porcine oropharynx for at least seven consecutive days. *S. suis* was also detected by PCR on all days despite the specific selection of *S. suis* negative piglets. This was also confirmed by MALDI-TOF analyses.

### Fever and pro-inflammatory cytokine responses

**Fig. 3:**
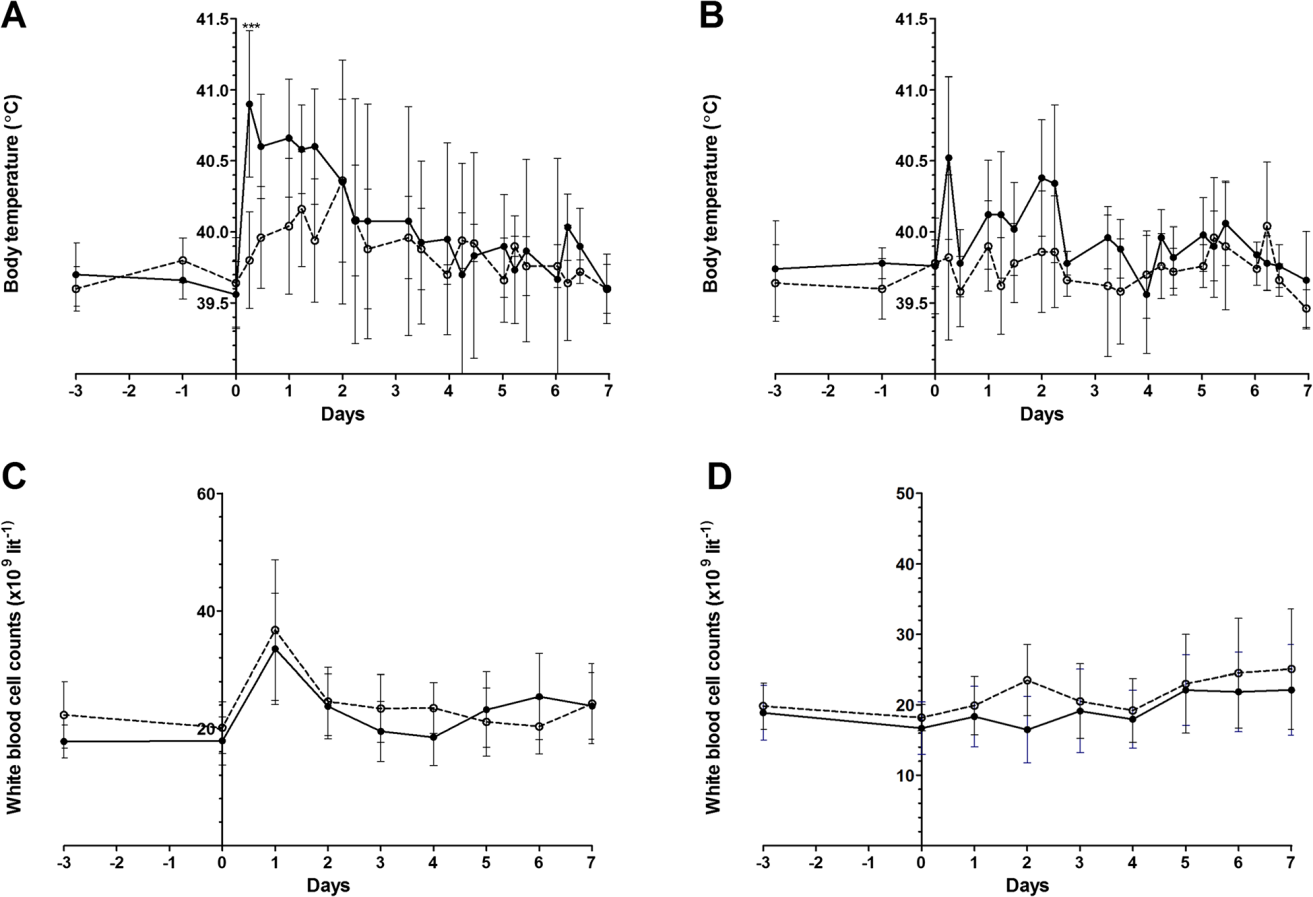
Body temperature and white blood cell counts of piglets infected with *S. pneumoniae*. Body temperature was recorded daily before inoculation and three times a day after inoculation. Mean body temperature of piglets inoculated intravenously (Panel **a**) or intranasally (Panel **b**) with *S. pneumoniae* are depicted. White blood cell counts were determined daily, mean counts are depicted for piglets inoculated intravenously (Panel **c**) or intranasally (Panel **d**). Open symbols represent piglets inoculated with a low dose, closed symbols piglets inoculated with a high dose. Each symbol represents 3 − 5 piglets, error bars show standard deviation. *** *p* < 0.001 (ANOVA)

**Fig. 4:**
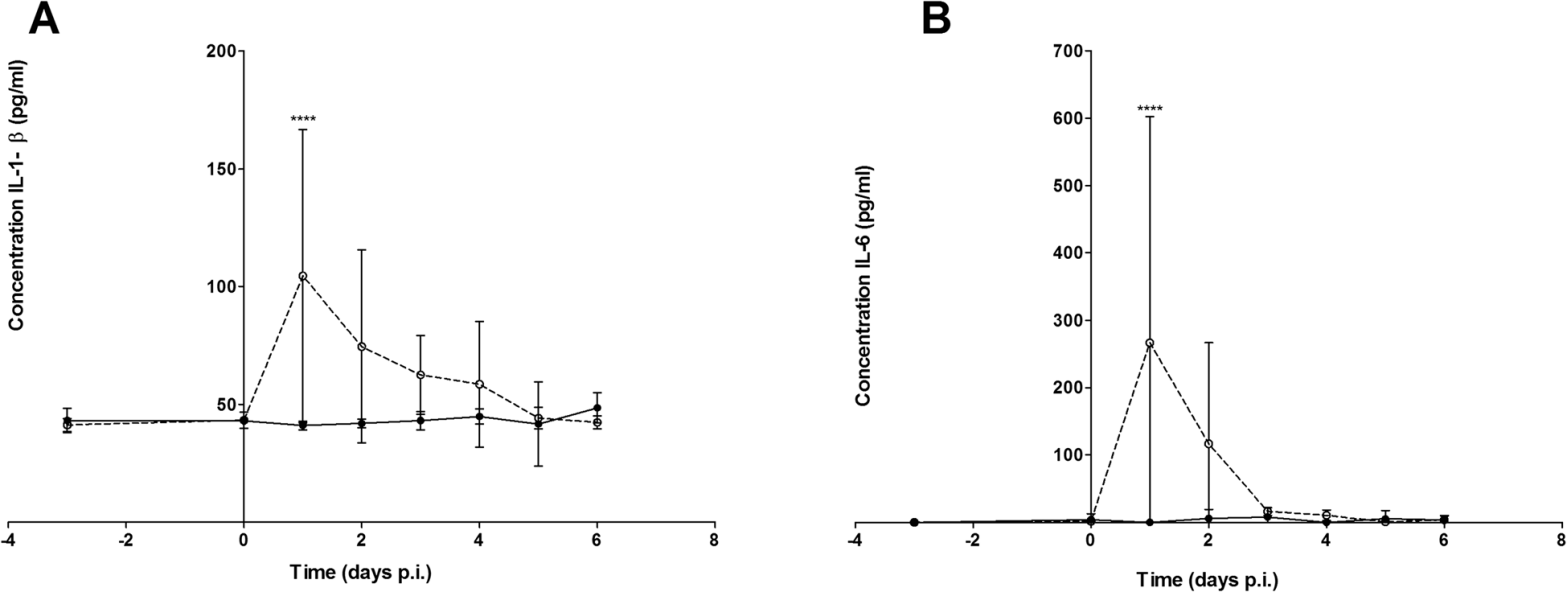
Systemic pro-inflammatory immune responses in piglets infected with *S. pneumoniae*. Serum concentrations of IL-1-β (panel **a**) and IL-6 (panel **b**) were determined by ELISA for piglets inoculated intravenously with a high dose of *S. pneumoniae* (open circles, dotted line) or intranasally with a high dose of *S. pneumoniae* (closed circles, solid line). Each symbol represents 3 – 5 piglets, error bars show standard deviation. *** *p* < 0.001 (ANOVA)

Immediately after inoculation an increase in body temperature (*p* < 0.01) was observed in all piglets inoculated with the high dose (intravenously as well as intranasally) (Fig. 3a and b). Piglets inoculated intravenously with a high dose were febrile from 6 h post-inoculation (p.i.) until 4 days p.i. and showed a second fever peak at 6 days p.i. Piglets inoculated intravenously with a low dose were only febrile from 24 h p.i. until 2 days p.i., but the temperature remained above pre-infection level for 5 days. The intranasally inoculated pigs displayed shorter febrile periods: the high dose group was febrile after 6 h p.i. until 2 days p.i., with a second peak at 5 days p.i. whereas the low dose group was only febrile once at 5 days p.i. One day after inoculation, white blood cell counts of the intravenously inoculated piglets increased significantly (p < 0.01), compared to white blood cell counts of the intranasally inoculated piglets, irrespective of the inoculation dose used (Fig. 3c and d). This leukocytosis is most likely the consequence of bacteraemia that was induced after intravenous inoculation. Systemic pro-inflammatory immune responses were monitored in both high dose groups of piglets. Intravenously infected piglets showed a peak production of Interleukin 6 (IL-6) and Interleukin 1 beta (IL-1-β) serum concentrations 1 day post-infection (p <0.001) (Fig. 4a and b) to decrease to normal levels respectively 3 and 5 days post-infection.

### Arthritis

**Fig. 5:**
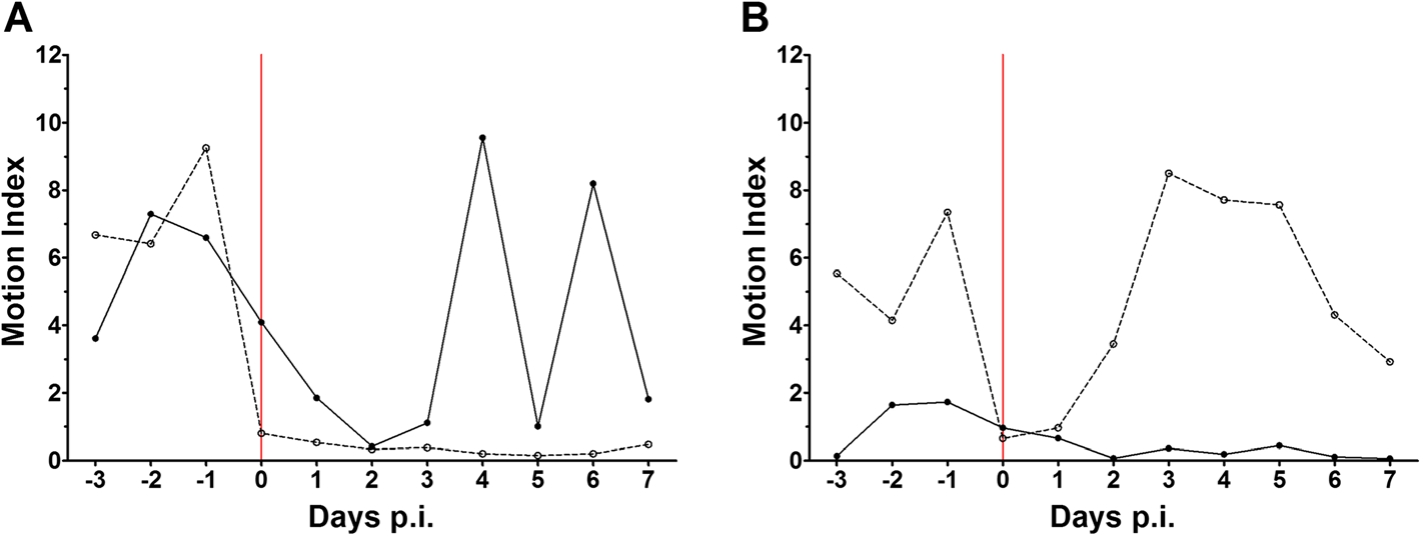
Playing behaviour of piglets during the experiment. Playing behaviour of piglets was measured in the intravenously infected piglets (panel **a**) and the intranasally infected piglets (panel **b**) using a pedometer attached to a hanging chain. Read out gives an estimation of playing behaviour for the whole group of piglets. Red line indicates the moment of infection. ● solid line indicates piglets infected with the high dose; ○ dashed line indicates piglets infected with the low dose

Four out of the five piglets inoculated intravenously with a high dose and one piglet inoculated intravenously with a low dose showed clinical signs of polyarthritis in several legs for one or more consecutive days (Table 1). Within 16 h after inoculation 4 piglets showed signs of arthritis (lameness, swelling of joints) in 1–3 legs, in one piglet arthritis in two joints was observed at 32 h post inoculation. In three piglets arthritis was short-lasting (less than 16 h); two piglets, both intravenously inoculated with a high dose were euthanized at 1 and 4 days post-infection as they reached the humane end points due to either the severity of disease in one piglet or the long duration of arthritis in another piglet. None of the piglets showed signs of arthritis from 4 days after inoculation on. Besides clinical manifestation of arthritis, no other signs of illness were observed at the regular clinical examination time points. However, immediately after inoculation, in all groups independent of the administration route or the inoculation dose used, the playing behaviour of piglets as measured by continuous recording of the movement of a toy chain, was considerably reduced compared to the pre-infection period (Fig. 5). These data clearly represent a discrete expression of subclinical illness after inoculation. At the time of necropsy, two piglets (one inoculated intravenously with a high dose and one inoculated intranasally with a low dose) showed signs of peritonitis with fibrinous exudates in the peritoneal cavity. Similar clinical signs of disease are frequently observed in piglets inoculated with *S. suis*, clearly indicating that *S. suis* as well as *S. pneumoniae* preferably cause infections at the serosae.

**Table 1:** Septic arthritis in piglets due to *Streptococcus pneumoniae*

Inoculation route	Maximum number of joints affected	Number of days with 1 or more arthritic joints
Intravenous	3	1^*^
	3	3^**^
	2	1
	2	1
Intranasal	1	1

^*^ Piglet was euthanized 1 day post-infection due to reaching humane end points

^**^ Piglet was euthanized 4 days post-infection due to reaching humane end points

### Pathology

**Fig. 6:**
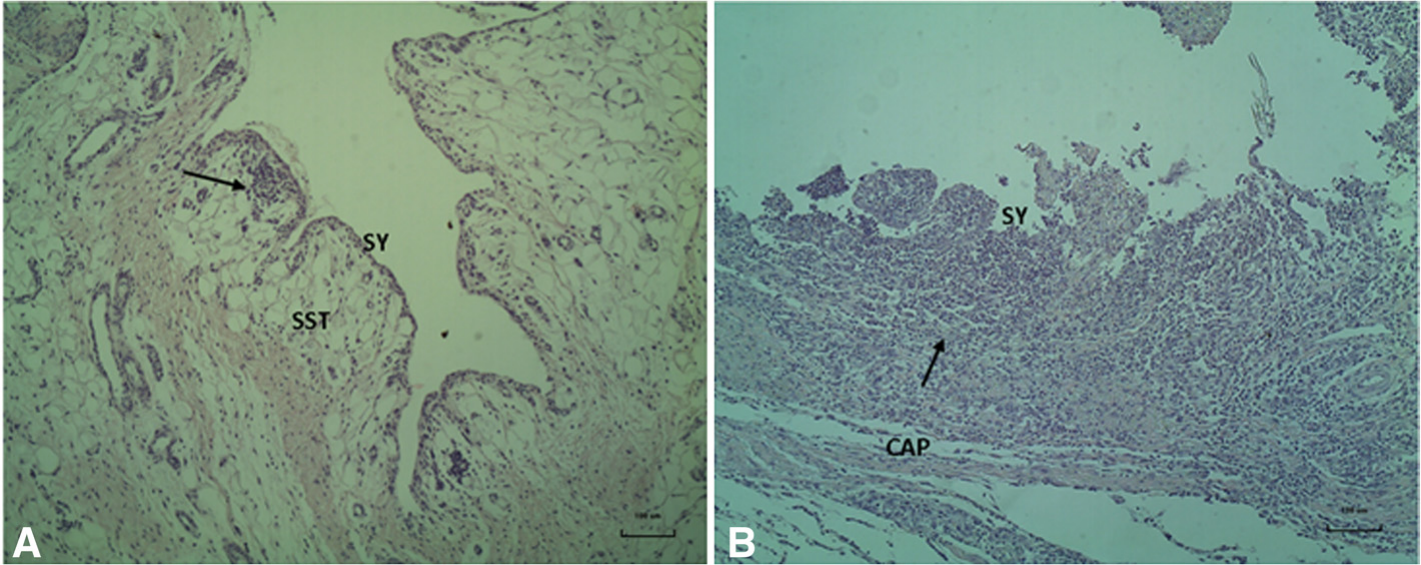
Histological findings in joints of piglets inoculated with *S. pneumoniae*. Panels **a** and **b** were obtained from synovial membranes (SY) of piglets inoculated intravenously with a high dose. *Panel* **a**: in joints with synovia hypersecretion typically few, small inflammatory foci with perivascular mononuclear cells (arrow) were seen in the subsynovial tissue (SST); *Panel* **b**: serofibrinous arthritis with disruption of the synovial membrane (SY) and extended inflammation of the subsynovial tissue (arrow) and the connective tissue (CT) of the joint capsule

During necropsy, five piglets inoculated intravenously (2 with the high dose and 3 with the low dose) as well as 2 piglets inoculated intranasally showed a serous hypersecretion of synovial fluid. Bacteriological examination of two joints with a serofibrinous arthritis revealed that the arthritis was caused by *S. pneumoniae*. Histological examination of the affected synovial membranes of the joints with arthritis revealed a serofibrinous synovialitis with moderate to extended mixed leucocyte cell infiltration into the subsynovial tissue in three piglets (Fig. 6b); in joints with increased synovial fluids the histological changes were characterized by few foci with perivascular mononuclear inflammatory cells in the subsynovial tissue (Fig. 6a). This strongly suggests that the clinical symptoms, overfilled joints and arthritis indeed were caused by a systemic pneumococcal infection. During gross pathology mild, focal abnormalities in lungs were observed in the intravenously inoculated low dose group as well as in both groups of intranasally inoculated piglets.

## Discussion

Here we demonstrate that pigs can be used as a model for human *S. pneumoniae* infections. Induction of pneumococcal bacteraemia results in persistent fever and disease, mainly septic arthritis. Systemic pro-inflammatory immune responses confirm the presence of pneumococcal induced inflammation in piglets. Moreover, we showed that *S. pneumoniae* is able to colonize the oropharyngeal cavity of piglets, allowing for colonization and transmission studies.

Stable oropharyngeal colonization of piglets with *S. pneumoniae* was determined by qPCR on tonsillar swab samples. Selective culturing of *S. pneumoniae* using gentamycin was not possible in pigs due to overgrowth of many different organisms, making it impossible to specifically count pneumococcal CFU. Despite the complex microbiome of porcine tonsils, consisting of more than 100 species [22, 23] including *S. suis*, it is apparently possible for *S. pneumoniae* to find its niche leading to colonization for at least 6 days. This enables the study of colonization as a function of time. Currently, many research groups concentrate on the development of a universal serotype-independent vaccine, based on pneumococcal proteins [24]. There is a lot of experience with vaccination challenge experiments in pigs with porcine pathogens with a similar pathogenesis as *S. pneumoniae* in humans, like *S. suis* [25, 26]. The piglet model described here may be an attractive alternative to study the efficacy of vaccine candidates, in particular at the level of colonization.

A significant number of intravenously inoculated piglets in this study developed arthritis. In humans [2] invasive disease caused by *S. pneumoniae* mainly leads to meningitis and sepsis. However, in humans *S. pneumoniae* can also cause septic arthritis [3]. Although human pneumococcal septic arthritis is less frequently observed it has been described as an emerging problem [27]. So far, an animal model inducing high levels of pneumococcal septic arthritis was not available.

Pneumococcal septic arthritis is often accompanied by a serum leukocytosis of > 11 × 10^9^ cells liter^−1^. Systemically infected piglets also showed a serum leukocytosis, where WBC counts > 34 × 10^9^ cells liter^−1^ were observed. The occurrence of arthritis after *S. suis* infection is very common in pigs, arthritis is often the first clinical symptom that is observed [28]. The different outcome of clinical disease caused by *S. pneumoniae* in humans and piglets might be due to the species barrier. Experimental *S. pneumoniae* models used doses ranging from 10^5^ in human colonization studies [19, 29] to 2 × 10^6^ in insects [30] and 5 × 10^6^ – 5 × 10^7^ in rodent models [31–33]. In this piglet model we used a dose of 2.5 × 10^6^ CFU (low) and 2.9 × 10^8^ CFU (high). The pneumococcal isolate used for intravenous infection in this study was obtained from a patient that suffered from pneumonia, meningitis and sepsis. Apparently in piglets the pathogenesis have steered towards arthritis, instead of to the development of meningitis. However, it cannot be excluded that another human pneumococcal isolate could also cause meningitis in piglets, with or without arthritis.

## Conclusion

Taken together, the data presented in the study demonstrate that central aspects of human pneumococcal infections can be modelled in piglets. Piglets can be very useful hosts for studies on mechanisms of colonization and invasive disease of *S. pneumoniae* infections as well as on the development of improved pneumococcal vaccines. Moreover this is the first example of an animal model inducing high levels of pneumococcal septic arthritis.

## Methods

### Infection

Twenty piglets (breed: Topigs 20) were purchased from a pre-screened farm with a defined microbiological porcine pathogen free status and were housed at the animal facilities of the Central Veterinary Institute with ad libitum access to water and feed. At the start of the experiment tonsil swabs of piglets were screened for the presence of *S. suis*. Animals negative for the most prevalent serotypes were selected. After an acclimatization period of 7 days, piglets were inoculated with *S. pneumoniae* at the age of 5 weeks, either intravenously (10 pigs) or intranasally (10 pigs), using two different doses. Intravenously inoculated piglets were injected with 1 ml of PBS containing either 4.2 × 10^6^ colony forming units (CFU) (low dose) or 2.9 × 10^8^ CFU (high dose) of *S. pneumoniae* strain PBCN214 into the vena jugularis. To establish colonization, piglets were inoculated intranasally by using 3 ml of PBS containing either 2.5 × 10^6^ CFU (low dose) or 2.9 × 10^8^ CFU (high dose) of *S. pneumoniae* BHN418 [29]. Therefore, aerosols of the inocula were produced by a commercial, gravity-fed, single trigger airbrush (Evolution™, Harder & Steenbeek) with a nozzle of 0.2 millimeter, creating an aerosol with about 10 % droplets which are smaller than 26 micrometer and about 50 % of droplets which are smaller than 50 micrometer in diameter.

### Monitoring the health status

To monitor the health status of piglets, body temperatures and clinical scores were systematically recorded 3 times a day, starting at 3 days before inoculation to record baseline levels. Swelling of joints, particularly carpal, tarsal and knee joints and occurring lameness were assessed and interpreted as arthritis. Pigs were restraint and blood was collected from the jugular vein on a daily basis starting 3 days before inoculation to monitor white blood cell counts (WBC), bacteraemia and to collect plasma. WBCs were counted using an automated cell counter (Sysmex, pocH-100iV-diff), including differentiation of blood cells.

### Monitoring infection by cultivation, MALDI-TOF and PCR analysis

Bacteraemia was monitored daily by plating 100 μl of ethylenediaminetetraacetic acid (EDTA) blood directly onto Columbia agar containing 5 % sheep blood and 5 μg ml^−1^ gentamycin. Plates were incubated for 16 h at 37 °C and colonies were counted. Bacteria isolated from blood were confirmed to be *S. pneumoniae* using the direct smear method on Bruker MALDI Biotyper Microflex V.3.1, with the Bruker taxonomy database (4613 entries). To obtain plasma, EDTA blood was centrifuged for 15 min at 2500 × *g*. Plasma was stored at −20 °C for subsequent analyses. Tonsil and nasal swabs were collected daily, starting from day 0, to monitor colonization of *S. pneumoniae*. Swabs were placed in 3 ml of sterile PBS containing 15 % glycerol and sonicated in a water bath (Ultrasonic Cleaner, VWR symphony) at room temperature for 90 min. to elute bacteria from the swabs. Subsequently, the material was stored at–20 °C for subsequent analyses.

### Necropsy

Animals were sacrificed at 7 days post-infection or when pigs reached predefined humane end points. All piglets were subjected to necropsy. During necropsy, samples from the nasal mucosa, tonsils, lungs at 6 locations, internal organs (peritoneum, liver, kidney, spleen, thorax, heart), and brain were taken for bacteriological examination. For this purpose, tissue was homogenized and serial dilutions were plated for enumeration. Affected organs were formalin fixed for histological examination. Formalin fixed organ material was embedded in paraffin, sectioned at 3 − 5 micrometer, and subsequently stained with haematoxylin and eosin. Sections were microscopically screened for pathological changes. Pro-inflammatory immune responses were determined using commercial Quantikine ® Enzyme-Linked Immunosorbent Assay (ELISA) kits for porcine Il-1-β and porcine IL-6 (R&D systems) according to instructions of the manufacturer.

### Quantification of pneumococcal DNA by qPCR

Pneumococcal DNA in tonsil and nasal swabs was determined by amplification of part of the autolysin (*lytA*) gene using qPCR (lytA-Fw: 5′-ACGCAATCTAGCAGATGAAGCA-3′; lytA-Rev: 5′-TCGTGCGTTTTAATTCCAGCT-3′). The 20 μl PCR mix consisted of 1× SsoAdvanced Universal SYBR Green Supermix (Biorad), 4 pmol of each primer, and 1 μl of the extracted DNA. Thermal cycling was performed in a CFX Real-time PCR system (Biorad) under the following cycling conditions: 3 min at 95 °C, and 40 cycles of 10 sec at 95 °C and 30 sec at 55 °C. A qPCR signal above 35 cycles was considered negative. Additionally the qPCR mix contained 4 pmol of probe 5′-(FAM)-GCCGAAAACGCTTGATACAGGGAG-(BHQI)-3′ [34]. A standard curve of a ten-fold dilution series of genomic DNA extracted from *S. pneumoniae* BHN418, extracted with Qiagen columns and quantified with a spectrophotometer (Nanodrop) was used to calculate the amount of pneumococcal DNA per swab.

## Abbreviations

(q) PCR, (quantitative) polymerase chain reaction; CFU, Colony forming units; EDTA, ethylenediaminetetraacetic acid; ELISA, enzyme-linked immunosorbent assay; IL-1, β–interleukin 1 beta; IL-6, interleukin 6; *lytA*, autolysin gene; MALDI-TOF, matrix-assisted laser desorption/ionization–time of flight; p.i. – post-inoculation; WBC, white blood cell counts
